# Supplementary material for: HIVprotI: an integrated web based platform for prediction and design of HIV proteins inhibitors
Source: J Cheminform. 2018 Mar 9;10:12. doi: 10.1186/s13321-018-0266-y (PMC5845081; doi:10.1186/s13321-018-0266-y)
Supplement: Supplementary file 2 — Additional file 2. Source code of HIVProtI web server. [file 13321_2018_266_MOESM2_ESM.zip › HIVprotI_Source-code/hivprotI/weka-3-6-6/doc/weka/classifiers/lazy/kstar/KStarCache.CacheTable.html]

KStarCache.CacheTable


---


|  |  |  |
| --- | --- | --- |
| **Overview** \| **Package** \| **Class** \| **Tree** \| **Deprecated** \| **Index** \| **Help** \| **Weka's home** | |  |
| **PREV CLASS**   **NEXT CLASS** | **FRAMES**    **NO FRAMES**     **All Classes** |  |
| SUMMARY: NESTED \| FIELD \| CONSTR \| METHOD | DETAIL: FIELD \| CONSTR \| METHOD |  |


---


## weka.classifiers.lazy.kstar Class KStarCache.CacheTable

```
java.lang.Object
  weka.classifiers.lazy.kstar.KStarCache.CacheTable
```

**All Implemented Interfaces:**: java.io.Serializable, RevisionHandler

**Enclosing class:**: KStarCache

---

``` public class KStarCache.CacheTable extends java.lang.Object implements java.io.Serializable, RevisionHandler ```

A custom hashtable class to support the caching system.

**See Also:**: Serialized Form

---

**Constructor Summary**

`KStarCache.CacheTable()`
  
          Constructs a new hashtable with a default capacity and load factor.

`KStarCache.CacheTable(int size,
float loadFactor)`
  
          Constructs a new hashtable with a default capacity and load factor.


| **Method Summary** | |
| --- | --- |
| `void` | `clear()`             Clears this hashtable so that it contains no keys. |
| `boolean` | `containsKey(double key)`             Tests if the specified double is a key in this hashtable. |
| `KStarCache.TableEntry` | `getEntry(double key)`             Returns the table entry to which the specified key is mapped in this hashtable. |
| `java.lang.String` | `getRevision()`             Returns the revision string. |
| `void` | `insert(double key, double value, double pmiss)`             Inserts a new entry in the hashtable using the specified key. |
| `boolean` | `isEmpty()`             Tests if this hashtable maps no keys to values. |
| `int` | `size()`             Returns the number of keys in this hashtable. |

**Methods inherited from class java.lang.Object**

`equals, getClass, hashCode, notify, notifyAll, toString, wait, wait, wait`

**Constructor Detail**

### KStarCache.CacheTable

```
public KStarCache.CacheTable(int size,
                             float loadFactor)
```

:   Constructs a new hashtable with a default capacity and load factor.

---


### KStarCache.CacheTable

```
public KStarCache.CacheTable()
```

:   Constructs a new hashtable with a default capacity and load factor.


**Method Detail**

### containsKey

```
public boolean containsKey(double key)
```

:   Tests if the specified double is a key in this hashtable.

---


### insert

```
public void insert(double key,
                   double value,
                   double pmiss)
```

:   Inserts a new entry in the hashtable using the specified key.
    If the key already exist in the hashtable, do nothing.

---


### getEntry

```
public KStarCache.TableEntry getEntry(double key)
```

:   Returns the table entry to which the specified key is mapped in
    this hashtable.

    :   **Returns:**: a table entry.

---


### size

```
public int size()
```

:   Returns the number of keys in this hashtable.

    :   **Returns:**: the number of keys in this hashtable.

---


### isEmpty

```
public boolean isEmpty()
```

:   Tests if this hashtable maps no keys to values.

    :   **Returns:**: true if this hastable maps no keys to values.

---


### clear

```
public void clear()
```

:   Clears this hashtable so that it contains no keys.

---


### getRevision

```
public java.lang.String getRevision()
```

:   Returns the revision string.

    :   **Specified by:**: `getRevision` in interface `RevisionHandler`
    :   **Returns:**: the revision


---


|  |  |  |
| --- | --- | --- |
| **Overview** \| **Package** \| **Class** \| **Tree** \| **Deprecated** \| **Index** \| **Help** \| **Weka's home** | |  |
| **PREV CLASS**   **NEXT CLASS** | **FRAMES**    **NO FRAMES**     **All Classes** |  |
| SUMMARY: NESTED \| FIELD \| CONSTR \| METHOD | DETAIL: FIELD \| CONSTR \| METHOD |  |


---
